# Reversible Changes in BDNF Expression in MK-801-Induced Hippocampal Astrocytes Through NMDAR/PI3K/ERK Signaling

**DOI:** 10.3389/fncel.2021.672136

**Published:** 2021-05-14

**Authors:** Wenjuan Yu, Hongwei Fang, Lei Zhang, Miaowen Hu, Sidi He, Huafang Li, Hao Zhu

**Affiliations:** ^1^Shanghai Mental Health Center, Shanghai Jiao Tong University School of Medicine, Shanghai, China; ^2^Department of Anesthesiology and Intensive Care Unit, Dongfang Hospital, Tongji University, Shanghai, China; ^3^Shanghai Clinical Research Center for Mental Health, Shanghai, China; ^4^Shanghai Key Laboratory of Psychotic Disorders, Shanghai, China; ^5^Department of Anesthesiology, Renji Hospital, Shanghai Jiao Tong University School of Medicine, Shanghai, China

**Keywords:** MK-801, Schizophrenia, astrocyte, BDNF, NMDA receptor

## Abstract

Dizocilpine (MK-801), a non-competitive N-methyl-D-aspartic acid receptor (NMDA-R) antagonist, can induce schizophrenia-like symptoms in healthy individuals, implicating NMDA-R hypofunction in disease pathogenesis. Brain-derived neurotrophic factor (BDNF) is also implicated in schizophrenia, and expression is regulated by NMDA-R activity, suggesting a functional link. We previously found that BDNF signaling was upregulated by MK-801 in cultured hippocampal astrocytes, but the underlying mechanism is not clear. To address this issue, the levels of BDNF expression and secretion were evaluated in hippocampal astrocytes incubated with MK-801 by ELISA and qPCR, with and without NMDA co-incubation or pretreatment of either the ERK1/2 inhibitor, PD98059 or the PI3K inhibitor, LY294002. The apoptosis, viability, and proliferation of the astrocytes were also examined. In the current study, we demonstrate that MK-801 treatment (20 μM for 5 days) enhances the proliferation of rat cultured hippocampal astrocytes. Expression of BDNF mRNA was enhanced after 24 h in MK-801, but returned to near baseline over the next 24 h in the continued presence of MK-801. However, two successive 24-h treatments enhanced BDNF expression. These application regimens had no effect on apoptosis or proliferation rate. Co-addition of NMDA significantly inhibited MK-801-induced upregulation of BDNF. Similarly, MK-801-induced BDNF upregulation was blocked by pretreatment with inhibitors of PI3K and ERK1/2, but not by inhibitors of p38 and JNK. These findings suggested that astrocytes may contribute to the acute neurological and behavioral response to MK-801 treatment via a transient increase in BDNF expression involving NMDA-R–PI3K–ERK signaling.

## Introduction

Astrocytes are the most abundant type of glial cell in the central nervous system, and crucial for neuroplasticity and neural homeostasis across life-span to modulate metabolic exchange by various secreted and contact-mediated signals (Clarke and Barres, 2013; Araque et al., 2014). Astrocytes are important participants in many aspects of brain development, function, and disease (Clarke and Barres, 2013). Astrocytes are increasingly involved in the pathophysiology of schizophrenia due to synaptic defects (Takahashi and Sakurai, 2013). Astrocytes synthesize and secrete a large number of cytokines regulating neuroplasticity and responding to injury, including brain-derived neurotrophic factor (BDNF) (Cardile et al., 2003; Hong et al., 2016; de Pins et al., 2019).

Brain-derived neurotrophic factor (BDNF) is a member of the neurotrophic factor family and crucial for neuronal survival (Barde et al., 1982). BDNF is the most abundant neurotrophin in the brain, with particularly high expression in the hippocampus, prefrontal cortex (PFC), and hypothalamus, where it supports a variety of functions, such as the regulation of neuronal morphology and synaptic plasticity (Han and Deng, 2018; De Vincenti et al., 2019). By interacting with tropomyosin receptor kinase B (TrkB) and p75 neurotrophin receptor (p75NTR), BDNF facilitates long-term potentiation (LTP) and long-term depression (LTD), respectively, two forms of synaptic plasticity that may mediate certain forms of learning (Woo et al., 2005; Yano et al., 2006; Lu and Martinowich, 2008). Thus, disruption of BDNF signaling may be responsible for the cognitive deficits exhibited by schizophrenia patients (Chen et al., 2020). Indeed, multiple studies have indicated that BDNF plays a key role in the pathophysiology of schizophrenia (Lu and Martinowich, 2008; Chen et al., 2020).

Schizophrenia is a severe, debilitating mental disorder including positive symptoms (hallucinations and delusions), negative symptoms (avolition and anhedonia), and cognitive dysfunction and affects approximately 1% of the global population (Capuano et al., 2002; Jobe and Harrow, 2005). Systemic administration of dizocilpine (MK-801), a non-competitive NMDA-R antagonist, can induce schizophrenia-like symptoms in rodents (Yu et al., 2011; Deiana et al., 2015), and acute MK-801 administration is now widely used as a model for studies on pathogenic mechanisms and antipsychotic drug efficacy for schizophrenia. Our previous study showed that repeated high doses of MK-801 (0.5 mg/kg every day for 6 days) activated primary cultured hippocampal astrocytes as evidenced by enhanced GFAP expression and upregulated BDNF expression at both the protein and mRNA levels (Yu et al., 2015). The astrocytes may play an important biological role in synaptic plasticity through autocrine and paracrine BDNF. However, the signaling mechanisms underlying this effect of MK-801 on BDNF expression in hippocampal astrocytes are unknown.

MK-801 can activate a variety of signaling pathway components in the rat brain, including the PI3K-Akt-GSK3β and MEK-ERK pathways (Ahn et al., 2005, 2006; Seo et al., 2007). It is widely known that NMDARs activate primary signaling cascades including PI3K and ERK signaling pathways, both of which are involved in the pathogenesis of schizophrenia and the therapeutic mechanisms of antipsychotic agents (Seo et al., 2007). PI3K is an essential upstream regulator of ERK activation, and it has been reported that the activation of ERK stimulated by NMDA receptor is completely or partially dependent on PI3K activity (Opazo et al., 2003).Therefore, the current study was designed to assess the contributions of various NMDA-R-linked signaling pathways to MK-801-induced upregulation of astrocytic BDNF expression and release.

## Experimental Procedures

### Primary Astrocyte Cultures

Astrocyte cultures were established as described in our previous study (Yu et al., 2015). Briefly, astrocyte cultures were prepared from the hippocampi of 2-days-old neonatal Sprague–Dawley rats following mechanical dissociation. Dissociated cells were suspended in Dulbecco’s Modified Eagle’s Medium (DMEM) (Gibco, Invitrogen, Grand Island, NY) supplemented with 10% fetal bovine serum (FBS; Gibco) and 1 mM glutamine (Gibco). Cells were seeded on uncoated 25-cm^2^ flasks at 200,000 cells/cm^2^. Medium was changed 2 days after seeding and twice weekly thereafter. When cultures reached confluence (10–11 days after plating), non-astrocytes such as microglia were detached from the flasks by shaking and the medium was replaced. Astrocytes were detached using 0.25% EDTA-trypsin (Sigma, St. Louis, MO, United States) and passaged. Experiments were initiated after the second passage.

Cultured astrocytes were identified through immunofluorescence. Cells were fixed with 4% paraformaldehyde and probed with rabbit anti-GFAP (1:500, Sigma, United States), then incubated with Cy3-conjugated goat donkeyanti-rabbit IgG (1:100, Abcam) for immunofluorescence. The nuclei were stained by Hoechst 33342. The results of immunostaining were examined with a Leica DM2500 microscope using a DDC 2/3 camera.

### Annexin V–FITC/Propidium Iodide Double-Staining Apoptosis Assay

Apoptosis rates were evaluated using the Annexin V-FITC Apoptosis Detection Kit (Thermo Fisher Scientific, United States). Cultured astrocytes were passaged and plated in 12-well plates at 5 × 10^5^ cells per well. The cells were treated with MK-801 ([5R, 10S]-[+]-5-methyl-10,11-dihydro-5H-dibenzo[a,d] cyclohepten- 5,10-imine; Sigma) for 48 h using two regimens, continuously without drug exchange (20 μM/48 h group) or two successive 24-h applications [(20 μM + 20 μM)/48 h group]. Treated astrocytes were then collected, washed twice in cold PBS with gentle shaking, and dispersed in 200 μL binding buffer solution containing 10 μL Annexin-FITC and 5 μL propidium iodide (PI). The binding reaction was allowed to proceed for 15μmin at room temperature in the dark. Apoptotic rate (proportion of Annexin-FITC/PI+ cells) was measured by flow cytometry (Beckman Coulter, Inc., Fullerton, CA, United States).

### Cellular Proliferation Assay

The effects of MK-801 on astrocyte proliferation were assessed by an ELISA-based BrdU incorporation assay (Roche Molecular Biochemicals, Mannheim, Germany) according to the manufacturer’s protocol. Cultured astrocytes were passaged and plated in 96-well plates at 5 × 10^4^ cells per well and left untreated (controls) or treated with 20 μM MK-801 for 24 h. The supernatant was then replaced with fresh medium containing the same MK-801 concentration and astrocytes were incubated for another 1, 2, or 4 days. Before the assay, BrdU was added at a final concentration of 10 μM. Cells were incubated for 2 h at 37°C, fixed with fixation solution for 30 min at room temperature, and incubated with 100 μL peroxidase-labeled anti-BrdU antibody for 90 min. After three washings, a substrate solution for colorimetric quantification was added at 100 μL/mL and cultures left at room temperature for 5–30 min until color development was sufficient for photometric detection. The reaction products were quantified by measuring the absorbance at 370 nm (reference wavelength, 492 nm) using a scanning multiwell spectrophotometer equipped with Gen 5 analysis software (Synergy HT multimode microplate reader, BioTek Instruments Inc., Bad Friedrichshall Germany). Absorbance is directly correlated with DNA synthesis and ensuing BrdU incorporation, and thus, provides an index of proliferating cell number.

### Real-Time Quantitative Reverse Transcription-Polymerase Chain Reaction (qPCR)

Cultured astrocytes were passaged, plated in 3.5 cm dishes at 1 × 10^6^ cells/dish, and treated with 20 μM MK801 for 0, 24, or 48 h. Time-matched untreated cultures served as controls. In a second set of experiments, three treatment groups were established: 20 μM MK-801 continuously for 48 h (20 μM/48 h group), two successive 24-h treatments with 20 μM MK801 [(20 μM + 20 μM)/48 h group], and untreated controls. In a third set of experiment, four treatment groups were established: 20 μM MK-801 for 24 h, 20 μM NMDA for 24 h, 20 μM MK-801 plus 20 μM NMDA for 24 h, and untreated controls. In a fourth set of experiment, astrocytes were treated directly with 20 μM MK-801 for 24 h or first pretreated with 20 μM of the ERK1/2 inhibitor PD98059 (Sigma), 20 μM of the PI3K inhibitor LY294002 (Sigma), 20 μM of the JNK inhibitor SP600125, or 20 μM of the p38 inhibitor SB203580 for 2 h prior to 20 μM MK-801 for 24 h. The mRNA levels in each treatment group were assessed by real-time quantitative PCR using a protocol described in our previous report (Gui et al., 2013). Briefly, total RNA from cultured astrocytes was extracted using TRIzol reagent (Invitrogen, Carlsbad, CA, United States) and reverse transcribed using the Perfect Real Time PrimeScript RT Reagent kit (Takara Bio, Otsu, Japan). Expression levels of BDNF and GAPDH (control) mRNA were quantified on a Light Cycler system (Roche, Indianapolis, IN, United States) using the QuantiTect SYBR Green PCR kit (Qiagen, Valencia, CA). The sequences of forward and reverse primers are listed in Table 1. The expression level of each gene was normalized to the mean Ct value of the housekeeping gene GAPDH in the PCR array. Differences in expression between treatment groups were determined by the 2^–ΔΔ*Ct*^ method. Each sample was analyzed in triplicate.

**TABLE 1:** The sequences of gene-specific primers used for qPCR.

**Gene name**	**Forward (5′–3′)**	**Reverse (5′–3′)**
BDNF	GTCACAGCGGCAGATAAAAAG	ATGGGATTACACTTGGTCTCGT
GAPDH	AGGGTGGTGGACCTCATGG	AGCAACTGAGGGCCTCTCTCTT

### MTT Assay

The MTT [3(4,5-dimethylthiazol-2-yl)2,5-diphenyltetrazolium bromide] assay was used to study the effect of MK801 on astrocyte viability. Cells in 96-well plates were pretreated with 20 μM PD98059, 20 μM LY294002, 20 μM SP600125, or 20 μM SB203580 for 2 h prior to incubation in 20 μM MK-801 for 24 h or treated with MK-801 alone without pretreatment. Cells were then washed in PBS and incubated in MTT stock solution (10 μL at 10 mg/mL) (Sigma) plus the remaining medium for another 4 h at 37°C. The medium was discarded after the incubation and the insoluble dark blue formazan formed by viable cells was dissolved in 100 μL DMSO. Formazan formation was quantified by absorbance at 570 nm with a reference wavelength of 630 nm using a microtiter plate reader (Bio-Rad Laboratories, Hercules, CA, United States). The viability of treated cultures was expressed relative to untreated controls (defined as 100%).

### Enzyme-Linked Immunosorbent Assay (ELISA)

Cultured astrocytes were passaged and plated in 3.5 cm dishes at 1 × 10^6^ cells/dish, then either incubated directly in 20 μM MK-801 for 24 h or first pretreated with 20 μM PD98059 (Sigma), 20 μM LY294002 (Sigma), 20 μM SP600125, or 20 μM SB203580 for 2 h prior to incubation in MK-801. The culture supernatants were collected and BDNF concentrations measured using a commercially available ELISA kit (BDNF Emax Immunoassay; Promega, Madison, WI, United States), according to the manufacturer’s instructions. Briefly, a monoclonal antibody was added to each well of 96-well plates followed by overnight incubation at 4°C. The following reagents were then sequentially added to the wells: samples and BDNF standards in duplicate (2 h at room temperature), anti-human BDNF polyclonal antibody (2 h at room temperature), anti-IgY horseradish peroxidase (1 h at room temperature), and 3,3’,5,5’-tetramethylbenzidine solution (20 min at room temperature). Plates were washed with Tris-buffered saline containing 0.05% Tween 20 and a stop solution was added to each well. The absorbance was measured at 450 nm using a microplate reader (Bio-Rad) within 30 min. BDNF concentration was calculated based on a standard curve.

### Statistical Analysis

Results are expressed as the mean ± SEM. The independent sample t test was used for two-group comparisons and one-way analysis of variance (ANOVA) followed by post hoc Newman–Keuls tests to compare multiple treatment groups. A *P* < 0.05 (two-tailed) was considered statistically significant.

## Results

### The MK-801 Dose Range Used Did Not Induce Apoptosis of Primary Hippocampal Astrocytes

**FIGURE 1:**
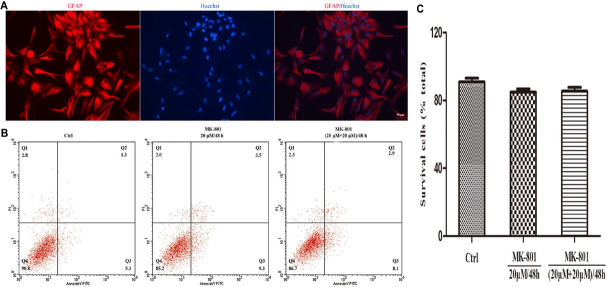
Immunofluorescence staining and apoptosis analysis of astrocytes. **(A)** GFAP positive cells (red) represent astrocytes, cell nuclei (blue) were stained with Hoechst33342, and the image showed high purity of astrocytes. Scale bar: 50 μm. **(B,C)** Primary hippocampal astrocytes were treated with 20 μM MK-801 continuously for 48 h (20 μM/48 h) or with two successive 24-h doses [(20 μM+20 μM)/48 h]. Untreated cultures served as controls (Ctrl). **(B)** Apoptosis was examined by Annexin V-FITC/PI using flow cytometry. The proportion of dead cells (Q1: Annexin V-FITC-/PI+), late apoptotic/necrotic cells (Q2: Annexin V-FITC+/PI+), early apoptotic cells (Q3: Annexin V-FITC+/PI-) and survival cells (Q4: Annexin V-FITC-/PI-) was displayed. **(C)** The equation (number of cells in Q4)/(total cell number) was used to calculate the survival cells rate. There were no significant differences in apoptosis rate among treatment groups. Data represent the mean ± SEM from three independent experiments. Statistical differences were assess by one-way ANOVA followed by Newman–Keuls multiple comparison tests.

Glial fibrillary acidic protein (GFAP) is expressed in astrocytes and used as a marker of astrocytes. GFAP was used to immunostain primary cultured hippocampal astrocytes (red) and Hoechst 33342 to stain nuclei (blue) (Figure 1A). Cells with merged blue nuclei and red cell bodies were identified as astrocytes. About 92% of cells in the primary culture were verified as astrocytes.

The effect of MK-801 on apoptosis rate was examined by Annexin/PI staining and flow cytometry. There was no significant increase in response to MK-801 treatment for 48 h compared to the control group, either when applied continuously or as two successive doses for 24 h (both *P* > 0.05, Figures 1B,C). Therefore, the MK-801 dose regimens used in this study are within the non-toxic range.

### Prolonged MK-801 Treatment Promoted Astrocyte Proliferation

**FIGURE 2:**
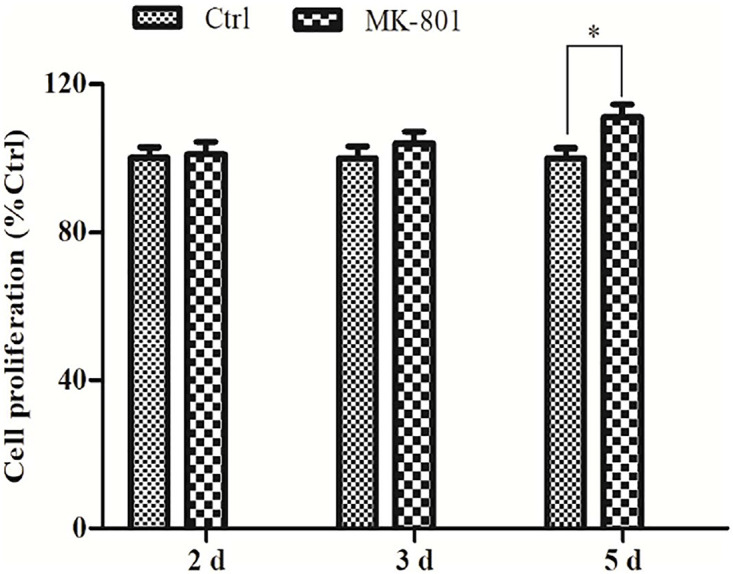
Prolonged MK-801 exposure increases astrocyte proliferation rate. Primary hippocampal astrocytes were treated with 20 μM MK-801 for 24 h, followed by medium exchange and continued incubation in MK-801 for another 1, 2, or 4 days. Cell proliferation was determined using an ELISA-based BrdU incorporation assay. A significant increase in proliferation was observed after 5 days of MK-801 treatment compared to control cultures. Data represent the mean ± SEM from three independent experiments. Statistical differences between paired groups were determined by independent samples *t*-test. ^∗^*P* < 0.05 vs. the untreated control group.

The effect of MK-801 on hippocampal astrocytes proliferation was determined using an ELISA-based BdrU incorporation assay. No significant differences were observed between the control and MK-801 group after 2 or 3 days in culture (Figure 2), but the BdrU signal was significantly greater in the MK-801 group after 5 days (111.2% ± 3.3% of control, *P* < 0.05, Figure 2).

### MK-801 Enhanced BDNF Expression by Hippocampal Astrocytes

**FIGURE 3:**
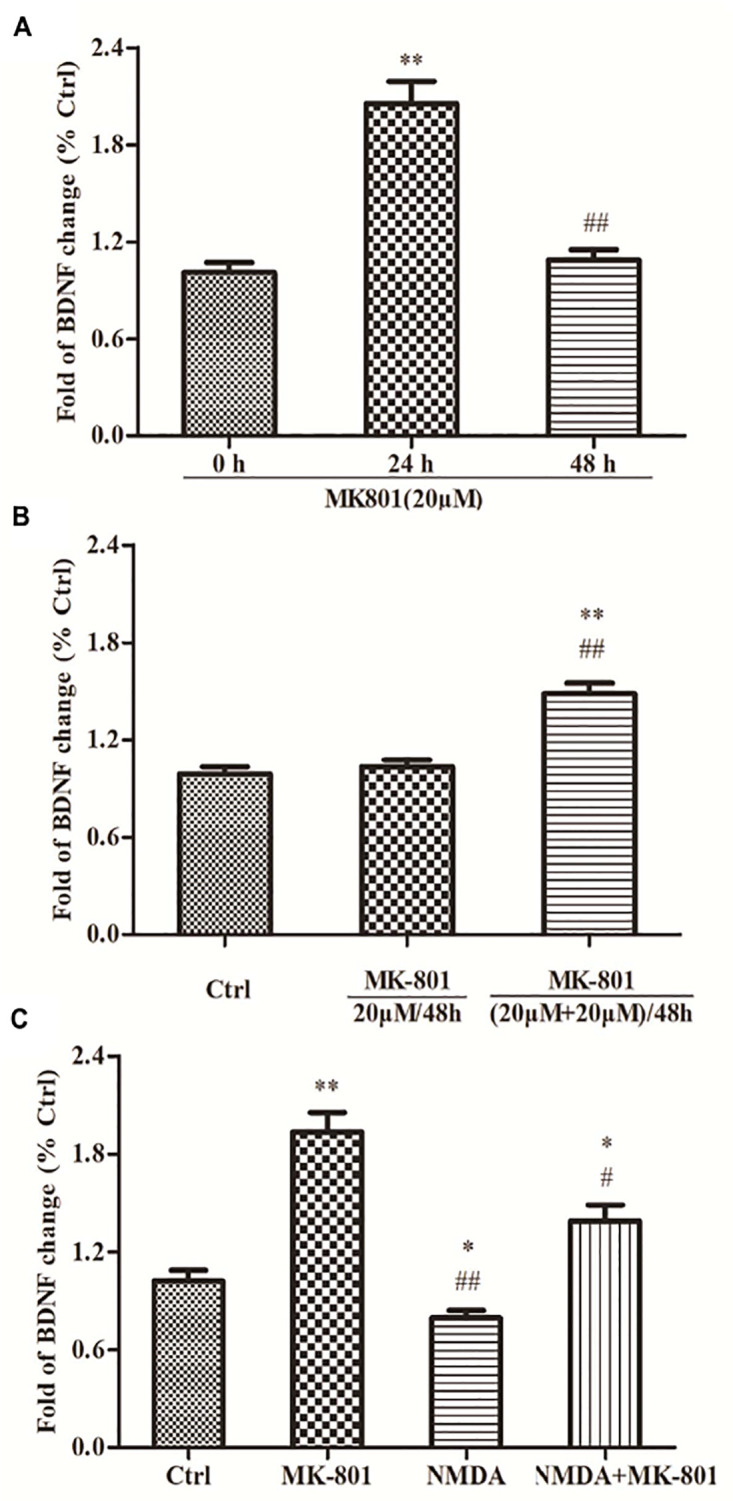
The expressions of BDNF mRNA in hippocampal astrocytes. **(A)** Primary hippocampal astrocytes were treated with 20 μM MK801 for 0, 24, or 48 h and BDNF mRNA expression was assessed by qPCR. Expression increased significantly after 24 h, but not after 48 h relative to untreated controls (0 h). **(B)** Two successive administrations of MK-801 for 24 h each also enhanced BDNF mRNA expression [(20 μM+20 μM)/48 h group] compared to untreated controls (Ctrl). **(C)** Primary hippocampal astrocytes were treated with 20 μM MK-801, 20 μM NMDA, or both for 24 h. mRNA levels were detected by qPCR. NMDA reduced basal BDNF mRNA expression and reversed the increase induced by MK-801. Expressed as the mean ± S.E.M of three independent experiments. Statistical differences between groups were determined by one-way ANOVA followed by Newman–Keuls multiple comparison tests. ^∗^*P* < 0.05 and ^∗∗^*P* < 0.01 vs. 0 h group or control group, ^#^*P* < 0.05 and ^ ##^*P* < 0.01 vs. 24 h group or MK801 20 μM/48 h group.

Astrocytes were incubated in 20 μM MK-801 and collected after 24 or 48 h for BDNF mRNA expression analysis using qPCR. BDNF mRNA expression was significantly increased after 24 h to approximately 2.1-fold of the control (0 h) group (*P* < 0.01; Figure 3A), but returned to near baseline (1.1-fold above control) after 48 h (*P* > 0.05; Figures 3A,B). Two successive applications of 20 μM MK-801 for 24 h ((20 mM + 20 mM)/48 h group) enhanced BDNF mRNA expression significantly (1.5-fold) compared to controls (*P* < 0.01; Figure 3B) and compared to cultures treated with MK-801 being expressed continuously for 48 h (*P* < 0.01; Figure 3B).

### NMDA Co-treatment Reversed the MK-801-Evoked an Increase in BDNF mRNA Expression by Hippocampal Astrocytes

Astrocytes treated with 20 μM MK-801, 20 μM NMDA, or both for 24 h were collected and BDNF mRNA expression evaluated using qPCR. BDNF mRNA expression was significantly elevated in the MK-801 group (*P* < 0.01; Figure 3C), but was reduced by NMDA compared (−20%) to untreated controls (*P* < 0.01; Figure 3C). Moreover, co-treatment with both MK-801 + NMDA significantly reversed the increase induced by MK-801 alone (*P* < 0.05; Figure 3C).

### MK-801 Enhanced Hippocampal Astrocytes Viability Through PI3K/ERK Signaling

**FIGURE 4:**
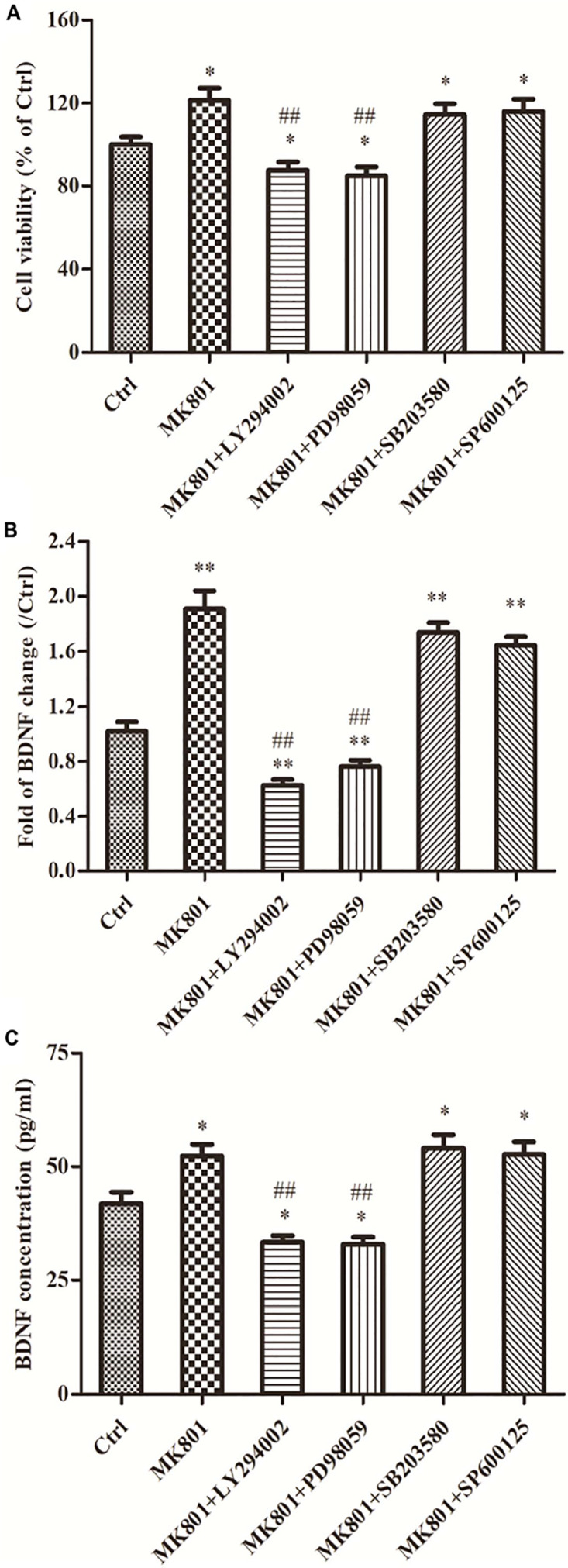
Signaling pathways are involved in cell viability, BDNF expression and secretion of hippocampal astrocytes incubated with MK-801. Primary hippocampal astrocytes were treated with 20 μM MK-801 alone for 24 h or pretreated with 20 μM ERK1/2 inhibitor PD98059, 20 μM PI3K inhibitor LY294002, 20 μM JNK inhibitor SP600125, or 20 μM p38 inhibitor SB203580 for 2 h prior to MK-801 treatment. **(A)** The viability of hippocampal astrocytes was determined by MTT assays. Both LY294002 and PD98059 (but not SB203580 or SP600125) completely reversed the enhanced viability induced by MK-801. **(B)** Expression of BDNF mRNA was assayed by qPCR. Both LY294002 and PD98059 significantly inhibited MK-801-induced BDNF upregulation in hippocampal astrocytes, while no significant changes were detected in SB203580 or SP600125 pretreatment groups compared to the MK-801 group. **(C)** Accumulation of BDNF in the culture supernatant was measured by ELISA assay. Pretreatment with LY294002 or PD98059 significantly inhibited BDNF secretion compared to MK-801 alone, while pretreatment with SB203580 or SP600125 had no effect. Values are expressed as mean ± S.E.M of three independent experiments. Statistical differences between groups were determined by one-way ANOVA followed by Newman-Keuls multiple comparison tests. ^∗^*P* < 0.05 and ^∗∗^*P* < 0.01 vs. control group, ^##^*P* < 0.01 vs. MK-801 group.

The effect of MK-801 on hippocampal astrocyte viability was assessed using an MTT reduction assay. After 24 h in 20 μM MK-801, the estimated number of viable astrocytes was 121.4% ± 5.9% of control (*P* < 0.05; Figure 4A). However, pretreatment with the ERK1/2 inhibitor PD98059 or the PI3K inhibitor LY294002 completely reversed this increase (to 84.8% ± 4.5 and 87.6% ± 4.0% of control, respectively, both *P* < 0.01 vs. MK-801 Figure 4A). Alternatively, viable cell count remained elevated following MK-801 treatment in cultures pretreated with the p38 inhibitor SB203580 or the JNK inhibitor SP600125 (both *P* > 0.05 vs. the MK-801 group; Figure 4A).

### MK-801 Promoted BDNF mRNA Expression in Hippocampal Astrocytes Through PI3K/ERK Signaling

As demonstrated by qPCR, the MK-801-induced increase in BDNF mRNA expression was complete reversed by LY294002 and PD98059 pretreatment (to 62.4 and 76.0% of control, respectively, both *P* < 0.01 vs. MK-801 alone; Figure 4B). Alternatively, BDNF mRNA expression levels were still elevated to 1.7-fold and 1.6-fold of the control in SB203580 and SP600125 pretreatment groups, respectively, and did not differ from the MK-801 group (both *P* > 0.05; Figure 4B).

### MK-801 Enhanced BDNF Protein Secretion in Hippocampal Astrocytes Through PI3K/ERK Signaling

The results of BDNF secretion assays using ELISA were consistent with expression results using qPCR. The BDNF concentration in culture supernatant was significantly elevated by MK-801 treatment compared to untreated controls (52.3 ± 2.6 vs. 41.9 ± 2.5 pg/mL; *P* < 0.05; Figure 4C) and this elevation was reversed by pretreatment with the PI3K inhibitor LY294002 or the ERK1/2 inhibitor PD98059 (33.3 ± 1.5 and 32.9 ± 1.6 pg/mL, respectively, both *P* < 0.01 vs. the MK-801 group; Figure 4C), but not by the p38 inhibitor SB203580 or JNK inhibitor SP600125 (54.1 ± 2.9 and 52.7 ± 2.7 pg/ml, respectively (both *P* > 0.05 vs. MK-801 alone; Figure 4C).

## Discussion

MK-801, a non-competitive NMDA receptor antagonist, is known to induce strong psychomimetic reactions such as hallucinations and psychomotor symptoms, and used widely in schizophrenia research. In the present study, we demonstrate that MK-801 can increase BDNF release from hippocampal astrocytes via ERK/PI3K signaling, which may contribute to these neurological and behavioral responses.

It is unlikely that non-specific deleterious effects of MK-801 contributed to our findings. There was no significant apoptosis in response to continuous MK-801 treatment for 48 h or two successive treatments for 24 h. In contrast, chronic MK-801 treatment of cultured astrocytes (>36 h) was reported to induce substantial cytotoxicity (Martins-de-Souza et al., 2011), but that study examined the human astrocytoma cell line 1321 N1 and used a slightly higher MK-801 concentration. Alternatively, no significant apoptosis of 1321 N1 cells was found in another study administering 50 μM MK-801 for 8 h (Guest et al., 2015).

In addition to enhanced BDNF secretion, prolonged MK-801 treatment (5 days) also promoted greater astrocyte proliferation as measured by BdrU incorporation. It is reasonable to speculate that MK-801-induced BDNF release eventually reaches a threshold to enhance proliferation rate. In addition, MK801 for as little as 24 h enhanced astrocyte viability, as measured by MMT assay. Ding et al. (2018) found that neural stem cell proliferation and viability were significantly reduced by MK-801 treatment for 24 h, but the concentration of MK-801 was high enough (200 μM) to induce significant apoptosis (Ding et al., 2018). Consistent with the present results, MK-801 enhanced expression of the astrocyte-specific intermediate filament protein GFAP, a marker of cell activation in response to local brain injury (Yu et al., 2015). Again, it is possible that BDNF release preserved cell viability via autocrine signaling.

Activated astrocytes alters the expression levels of molecules related to metabolism, neurotransmitters release, neuroplasticity, and neuron–glial signaling. Reactive astrocytes communicate with neurons via extracellular chemical signals, including neurotrophic factors such as BDNF (Koyama et al., 2003). BDNF expressions in the astrocytes are very low in the unlesioned adult brain, but increased in response to physiological and pathological neuronal signals, a form of neuron–glial signaling regulating neuroplasticity and enhancing neuronal resistance to injury (Parpura and Zorec, 2010; Fulmer et al., 2014). Indeed, astrocyte-derived BDNF was shown to reversed deficit following cuprizone-induced demyelination (Fulmer et al., 2014) and promote oligodendrogenesis during endogenous recovery from white matter damage (Miyamoto et al., 2015).

This BDNF increase was transient under our experimental conditions, in agreement with several previous studies. For instance, the acute neurochemical effects of single-dose MK-801 have been found to be reversible within 18–24 h over the dose range 0.3–1.0 mg/kg body weight (Olney et al., 1989; Horváth et al., 1997; Zou et al., 2019). Another study reported that 2 mg/kg MK-801 transiently enhanced MEK-ERK phosphorylation (Ahn et al., 2009). Furthermore, impaired locomotion, enhanced sniffing behavior, and increased ataxia were evident immediately after a single systemic injection of MK801 (5 mg/kg), but disappeared 24 h after treatment (Manahan-Vaughan et al., 2008). Repeated MK-801 injection at 0.5 mg/kg body weight had no cumulative neurotoxic effects when the treatments were given 24 h apart (Olney et al., 1989). A robust, dose- and time-dependent increase in phospho-ERK immunoreactive neurons was observed within the medial prefrontal cortex (mPFC) and central nucleus of the amygdala induced by a single dose of ketamine, another non-competitive NMDA receptor antagonist (Ardalan et al., 2020). Finally, a single dose of NMDA receptor antagonist, including MK801, induced acute and short-lasting behavioral alterations in rats (Manahan-Vaughan et al., 2008), and it was speculated that astrocytes may be involved via a marked, transient increase in BDNF expression.

MK-801 binds to several PCP binding sites inside the receptor ion channel to induce dose-dependent and reversible depolarization of astrocytes (Longuemare et al., 1996). In turn, astrocytic depolarization may activate voltage-sensitive L-channels, leading to a rise in free cytosolic Ca^2+^ ([Ca^2+^]i) (Zhu et al., 2012). MK-801 has a favorable profile compared to other NMDA receptor antagonists because of its extremely high affinity (Kornhuber and Weller, 1997) and selectivity for the receptor PCP binding site (Wong et al., 1986). Therefore, MK-801 may upregulate BDNF expression in hippocampal astrocytes by inducing NMDAR hypofunction and altering [Ca^2+^] homeostasis. However, NMDA-R subunits in glial cells have characteristics different from neurons, including the lack of Mg^2+^ block and reduced Ca^2+^ permeability (Dzamba et al., 2013). Glutamate-induced Ca^2+^ influx increases are inhibited by MK-801 and the selective NR2B antagonists ifenprodil and Ro25-6981 in neurons, but not in astrocytes (Kato et al., 2006; Montes de Oca Balderas and Aguilera, 2015). Furthermore, AMPA/kainate receptors and mGluRs may play a key role in glutamate-induced [Ca^2+^]i increases in astrocytes (Kato et al., 2006). These studies suggest that NMDA receptors in the astrocytes function in a non-canonical, Ca^2+^ flux-independent manner. Thus, BDNF upregulation and signaling induced by MK-801 may not be involved in NMDAR-dependent calcium influx in astrocytes.

In our study, inhibitors of PI3K and ERK1/2, but not p38 or JNK signaling blocked MK-801-induced enhancement of BDNF expression in primary hippocampal astrocytes. It is widely known that NMDARs activate primary signaling cascades including PI3 kinase-Akt-GSK-3β and Ras-MAPK kinase (MEK)-MAPK signaling pathways, both of which are involved in the pathogenesis of schizophrenia and the therapeutic mechanisms of antipsychotic agents (Seo et al., 2007). The MAPKs consist of multiple subfamilies of serine/threonine protein kinases, the extracellular signal-regulated kinases 1 and 2 (ERK1/2), the c-Jun NH2-terminal kinases (JNK), and the p38 kinases, which collectively promote a large diversity of cellular functions in many cell types (Rai et al., 2019). A single treatment with MK-801 at 1 mg/kg induced acute phosphorylation of Akt-GSK-3β and MEK-ERK signaling pathway components (Ahn et al., 2005, 2006), but no effect on the phosphorylation of JNK, a kinase associated with the cellular stress response (Ahn et al., 2009). Repeated injection with MK-801 (0.5–2 mg/kg) also enhanced the phosphorylation of ERK and AKT pro-survival signaling pathways, but had no change in JNK and even downregulated c-Jun (Ahn et al., 2009) and reduced neuronal damage in the rat frontal cortex (Seo et al., 2007). In addition, a single dose of ketamine also produced a robust increase in ERK phosphorylation in the brain (Ardalan et al., 2020). Direct infusion of PCP significantly increased MEK-ERK activity in the brains of rats, but had no effect on JNK and p38 activities in all investigated brain regions, including hippocampus (Kyosseva et al., 2001). Further, MK-801 was shown to upregulate ERK at least in part by suppressing NMDA receptors (Guan et al., 2013).

PI3-kinase is an essential upstream regulator of ERK activation, and it has been reported that the activation of ERK stimulated by NMDA receptor is completely or partially dependent on PI3K activity (Opazo et al., 2003). The PI3-kinase is required to advance NMDA receptor signals to ERK, but not to JNKs, which suggests that PI3K may function as a gatekeeper to control NMDA-R signals to either the ERK or JNK pathway (Wang et al., 2007). It is widely accepted that PI3K–Akt and MEK–ERK pathways are the principal pathways contributing to neuronal survival (Hetman and Gozdz, 2004), whereas JNK/SAPK (stress activated protein kinase) and p38 MAPK promote cell death (Subramaniam and Unsicker, 2006). Administration of MK-801 to rat dams downregulated ERK mRNA in hippocampus of 1-month-old and juvenile offspring exposed to prenatal stress (Guan et al., 2013). Furthermore, MK-801 reduced electroconvulsive shock-induced phosphorylation of p38 and its upstream kinase MKK6 (Haddad, 2005). In addition, MK-801 protected against retinal ganglion cells apoptosis and attenuated p38 activation (Haddad, 2005).

However, there are also studies reporting that MK-801 can reduce ERK phosphorylation (Ahn et al., 2006). Thus, NMDA-Rs appear to activate opposing stimulatory and inhibitory pathways that mediated bidirectional control of ERK activity. The predominant pathway appears to depend on the strength of activation, with strong activation reducing ERK phospho-activation (rather than enhancing dephosphorylation) and weaker stimulation promoting ERK activation (Chandler et al., 2001). This suggests that different doses of MK801 may have different effects on ERK signaling. In the aforementioned study (Chandler et al., 2001), ERK phosphorylation decreased in response to 0.25–1 mg/kg MK-801 injection compared to vehicle treatment, but increased in response to 2 mg/kg and decreased again in response to 4–8 mg/kg (Ahn et al., 2006).

Repeated injection with 0.5, 1, and 2 mg/kg MK-801 also enhanced the phosphorylation of MEK–ERK pathway components and concomitantly enhanced CREB phosphorylation in the rat frontal cortex (Ahn et al., 2005). In addition, MK-801 prevented the decreased hippocampal CREB mRNA observed in the frontal cortex of offspring subjected to prenatal stress (Guan et al., 2013). Active ERK activates downstream transcription pathways by phosphorylating CREB-binding protein at Ser301 (Wang et al., 2007), and many CREB target genes encode key proteins related to neuronal plasticity, including BDNF (Wang et al., 2016).

In conclusion, astrocytes respond to injury or stress by enhancing expression of several proteins involved in schizophrenia. Our findings provided direct evidence that NMDA-R hypofunction may transiently enhance BDNF expression in hippocampal astrocytes through NMDAR/PI3K/ERK signaling. Collectively, these findings suggest that altered astrocytic BDNF signaling to hippocampal neurons may contribute to the acute behavioral states induced by NMDA-R antagonists. Additional studies are currently being performed at our institute to further elucidate the involvement of astroglial factors in the NMDA-R antagonist animal model of schizophrenia.

## Data Availability Statement

The original contributions presented in the study are included in the article/Supplementary Material, further inquiries can be directed to the corresponding author/s.

## Ethics Statement

The animal study was reviewed and approved by the Shanghai Jiao Tong University School of Medicine.

## Author Contributions

WY and HZ designed the experiments, wrote and edited the manuscript. HF, LZ, MH, and SH performed experiments and analyzed data. HL supervised the experiments. All authors contributed to the article, approved the submitted version and approved it for publication.

## Conflict of Interest

The authors declare that the research was conducted in the absence of any commercial or financial relationships that could be construed as a potential conflict of interest.
