# Supplementary material for: Reversible Changes in BDNF Expression in MK-801-Induced Hippocampal Astrocytes Through NMDAR/PI3K/ERK Signaling
Source: Front Cell Neurosci. 2021 May 14;15:672136. doi: 10.3389/fncel.2021.672136 (PMC8160225; doi:10.3389/fncel.2021.672136)
Supplement: Supplementary file 1 [file Data_Sheet_1.docx]

Supplementary Materials

Glial fibrillary acidic protein (GFAP) is expressed in astrocytes and used as a marker of astrocytes. GFAP was used to immunostain primary cultured hippocampal astrocytes (red) and Hoechst 33342 to stain nuclei (blue) (SFig.1). Cells with merged blue nuclei and red cell bodies were identified as astrocytes. About 92% of cells in the primary culture were verified as astrocytes.


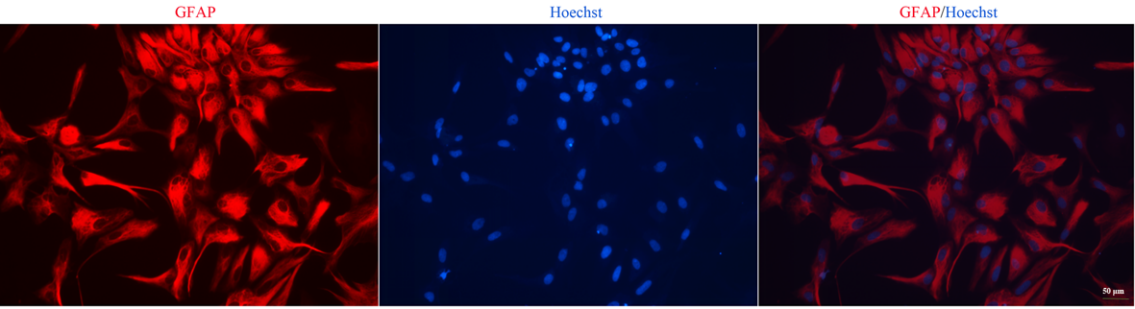


SFig. 1. Immunohistochemistry of primary cultured hippocampal astrocytes.

Astrocyte apoptosis was detected by flow cytometry. Primary hippocampal astrocytes were treated with 20 μM MK-801 continuously for 48 h (20 μM/48 h) or with two successive 24-h doses ((20 μM+20 μM)/48 h). Untreated cultures served as controls (Ctrl). Apoptosis was examined by Annexin/PI staining and flow cytometry. The data of survival cells ratio is in Table S1.

Table S1. The data of survival cells ratio

| Ctrl | MK801(20 μM/48 h) | MK801((20 μM+20 μM)/48 h) |
| --- | --- | --- |
| 94.5 | 89.5 | 91.7 |
| 91.8 | 81.2 | 85.9 |
| 86.2 | 84.8 | 82.6 |

Primary hippocampal astrocytes were treated with 20 μM MK-801 for 24 h, followed by medium exchange and continued incubation in MK-801 for another 1, 2, or 4 days. Cell proliferation was determined using an ELISA-based BrdU incorporation assay. The data of astrocyte proliferation is in Table S2.

Table S2. The data of astrocyte proliferation

| 2 d | | 3 d | | 5 d | |
| --- | --- | --- | --- | --- | --- |
| Ctrl | MK801 | Ctrl | MK-801 | Ctrl | MK-801 |
| 113.6 | 91.2 | 98.3 | 110.5 | 93.6 | 121.4 |
| 98.1 | 99.2 | 90.1 | 96.6 | 109.5 | 111.3 |
| 89.4 | 109.7 | 105.6 | 103.5 | 104.8 | 118.7 |
| 104.3 | 107.6 | 114.5 | 117.2 | 101.5 | 115.6 |
| 102.2 | 92.3 | 98.7 | 92.1 | 98.7 | 126.2 |
| 96.5 | 115.4 | 101.3 | 107.4 | 96.0 | 106.5 |
| 86.7 | 103.5 | 111.4 | 106.8 | 88.7 | 101.3 |
| 101.8 | 85.6 | 96.4 | 113.7 | 95.1 | 103.2 |
| 109.3 | 106.1 | 84.8 | 88.2 | 113.2 | 96.8 |

Primary hippocampal astrocytes were treated with 20 μM MK801 for 0, 24, or 48 h and BDNF mRNA expression was assessed by RT-PCR. Time-matched untreated cultures served as controls. The data of mRNA expression levels of BDNF are in Table S3.

Table S3. The data of BDNF mRNA expression levels

| 0 h | 24 h | 48 h |
| --- | --- | --- |
| 0.917462 | 1.853516 | 0.989521 |
| 0.981369 | 2.313412 | 1.210162 |
| 1.131581 | 1.997253 | 1.068075 |

Three treatment groups were established: 20 μM MK-801 continuously for 48 h (20 μM/48 h group), two successive 24-h treatments with 20 μM MK801 ((20 μM + 20 μM)/48 h group), and untreated controls. BDNF mRNA expressions were assessed by RT-PCR. The data of mRNA expression levels of BDNF are in Table S4.

Table S4. The data of BDNF mRNA expression levels

| Ctrl | MK-801(20 μM/48 h) | MK-801((20 μM+20 μM)/48 h) |
| --- | --- | --- |
| 0.920746 | 0.968956 | 1.603484 |
| 0.981369 | 1.110145 | 1.467822 |
| 1.071773 | 1.026430 | 1.382657 |

Primary hippocampal astrocytes were treated with 20 μM MK-801, 20 μM NMDA, or both for 24 h and mRNA levels were detected by RT-PCR. The data of mRNA expression levels of BDNF are in Table S5.

Table S5. The data of BDNF mRNA expression levels

| Ctrl | MK-801 | NMDA | NMDA+MK-801 |
| --- | --- | --- | --- |
| 1.129228 | 1.715371 | 0.8173364 | 1.580723 |
| 1.030000 | 2.123441 | 0.7055015 | 1.320043 |
| 0.912638 | 1.963868 | 0.8624698 | 1.266270 |

Primary hippocampal astrocytes were treated with 20 μM MK-801 alone for 24 h or first pretreated with 20 μM ERK1/2 inhibitor PD98059, 20 μM PI3K inhibitor LY294002, 20 μM JNK inhibitor SP600125, or 20 μM p38 inhibitor SB203580 for 2 h prior to MK-801 treatment. The viability of hippocampal astrocytes was determined by MTT assays. The data of astrocyte viability is in Table S6.

Table S6. The data of astrocyte viability

| Ctrl | MK801 | MK801  +LY294002 | MK801  +PD98059 | MK801  +SB203580 | MK801  +SP600125 |
| --- | --- | --- | --- | --- | --- |
| 102.6 | 124.3 | 92.3 | 92.1 | 112.3 | 113.4 |
| 105.8 | 141.7 | 103.4 | 76.8 | 123.6 | 104.7 |
| 96.4 | 116.7 | 79.5 | 101.6 | 104.9 | 95.2 |
| 92.2 | 109.4 | 88.2 | 80.5 | 97.1 | 125.8 |
| 113.7 | 132.8 | 75.6 | 71.4 | 117.7 | 134.7 |
| 89.6 | 103.2 | 86.7 | 86.4 | 131.6 | 122.3 |

Primary hippocampal astrocytes were treated with 20 μM MK-801 for 24 h or pretreated with 20 μM ERK1/2 inhibitor PD98059, 20 μM PI3K inhibitor LY294002, 20 μM JNK inhibitor SP600125, or 20 μM p38 inhibitor SB203580 for 2 h prior to MK-801. Expression of BDNF mRNA was assayed by RT-PCR. The data of mRNA expression levels of BDNF are in Table S7.

Table S7. The data of BDNF mRNA expression levels

| Ctrl | MK801 | MK801  +LY294002 | MK801  +PD98059 | MK801  +SB203580 | MK801  +SP600125 |
| --- | --- | --- | --- | --- | --- |
| 1.103357 | 2.167378 | 0.7039978 | 0.8469548 | 1.632355 | 1.751332 |
| 0.883749 | 1.803086 | 0.6124077 | 0.7485945 | 1.865914 | 1.531801 |
| 1.071147 | 1.752185 | 0.5558028 | 0.6852318 | 1.715439 | 1.643538 |

Primary hippocampal astrocytes were treated directly with 20 μM MK-801 or pretreated with 20 μM ERK1/2 inhibitor PD98059, 20 μM PI3K inhibitor LY294002, 20 μM JNK inhibitor SP600125, or 20 μM p38 inhibitor SB203580 for 2 h prior to MK-801. Accumulation of BDNF in the culture supernatant was measured by ELISA assay. The data of secretion levels of BDNF are in Table S8.

Table S8. Secretion levels of BDNF (pg/ml)

| Ctrl | MK801 | MK801  +LY294002 | MK801  +PD98059 | MK801  +SB203580 | MK801  +SP600125 |
| --- | --- | --- | --- | --- | --- |
| 46.3 | 51.2 | 31.2 | 35.8 | 54.9 | 54.5 |
| 37.6 | 48.5 | 36.1 | 32.6 | 48.6 | 56.3 |
| 41.7 | 57.3 | 32.7 | 30.3 | 58.7 | 47.2 |
